# Correction: H_2_-driven xylitol production in *Cupriavidus necator* H16

**DOI:** 10.1186/s12934-025-02655-7

**Published:** 2025-01-19

**Authors:** Tytti Jämsä, Nico J. Claassens, Laura Salusjärvi, Antti Nyyssölä

**Affiliations:** 1 VTT Technical Research Centre of Finland Ltd., Tekniikantie 21, 02150 Espoo, Finland; 2 Laboratory of Microbiology, Wageningen University, Stippeneng 4, 6708WE Wageningen, The Netherlands

**Correction: Microbial Cell Factories (2024) 23:345**

10.1186/s12934-024-02615-7

In the methods section, the phrase “flushing with 100% H2 at 0.5 mL/min for 2 min.” should read “flushing with 100% H2 at 0.5 L/min for 2 min.”

