# Supplementary material for: H2-driven xylitol production in Cupriavidus necator H16
Source: Microb Cell Fact. 2024 Dec 23;23:345. doi: 10.1186/s12934-024-02615-7 (PMC11665087; doi:10.1186/s12934-024-02615-7)
Supplement: Supplementary file 1 — Additional file 1: Table S1. Oligonucleotide primers used in the study. Table S2. Synthesized xylose reductase gene used in this study originating from Scheffersomyces stipitis. Table S3. Upstream and downstream regions of A0006 used to create C. necator H16 △A0006 . Figure S1. The growth of C. necator strains △phaCAB and △phaC on different sugars and sugar alcohols (100 mM). Figure S2. Comparison of bioconversion with a single H2 flush at the start and H2 flush after every sampling. Figure S3. SDS-PAGE analysis of soluble extracts by C. necator H16 strains. Full calculations. [file 12934_2024_2615_MOESM1_ESM.docx]

**Additional file 1**

**H_2_-driven xylitol production in *Cupriavidus necator* H16**

**Tytti Jämsä^1,*^, Nico J. Claassens^2^, Laura Salusjärvi^1^, Antti Nyyssölä^1^**

^1^ VTT Technical Research Centre of Finland Ltd., Tekniikantie 21, 02150 Espoo, Finland

^2^ Laboratory of Microbiology, Wageningen University, Stippeneng 4, 6708WE Wageningen, the Netherlands

*Correspondence to Tytti Jämsä: [tytti.jamsa@vtt.fi](mailto:tytti.jamsa@vtt.fi)

**Table S1**. Oligonucleotide primers used in the study.

| **Primer** | **5’ to 3’ sequence** | **Details** |
| --- | --- | --- |
| oXR1 | CCCAAGCTTTTAAAAGGGGAGGTATTTCTATGCCGTCGATCAAGCTGAA | PCR of synthesized *S. stipitis* XR for cloning |
| oXR2 | CTAGACTAGTACCCCTTCCTTACACGAAGA | PCR of synthesized *S. stipitis* XR for cloning |
| oSEVA_F | TCTAGGGCGGCGGAT | Colony PCR |
| oSEVA_R | CTGGATTCTCACCAATAAAAAACG | Colony PCR |

**Table S2.** Synthesized xylose reductase gene used in this study originating from *Scheffersomyces* *stipitis*. Codon-optimized for *C. necator* (957 bp). The original sequence is associated with GeneID 4839234.

| **Name** | **Nucleotide sequence** |
| --- | --- |
| XRsti | ATGCCGTCGATCAAGCTGAACTCGGGCTACGACATGCCGGCCGTGGGCTTCGGCTGCTGG  AAGGTGGACGTGGACACCTGCTCGGAACAGATCTACCGCGCCATCAAGACCGGCTACCGC  CTGTTCGACGGCGCCGAGGACTACGCCAACGAAAAGCTGGTGGGCGCCGGCGTGAAGAAG  GCCATCGACGAGGGCATCGTGAAGCGCGAAGACCTGTTCCTGACCTCGAAGCTGTGGAAC  AACTACCACCACCCGGACAACGTGGAGAAGGCCCTGAACCGCACCCTGAGCGACCTGCAG  GTGGACTACGTGGACCTGTTCCTGATCCACTTCCCGGTGACCTTCAAGTTCGTGCCGCTG  GAGGAAAAGTACCCGCCGGGCTTCTACTGCGGCAAGGGCGACAACTTCGACTACGAGGAC  GTGCCGATCCTGGAAACCTGGAAGGCCCTGGAGAAGCTGGTGAAGGCCGGCAAGATCCGC  TCGATCGGCGTGTCGAACTTCCCCGGCGCCCTGCTGCTGGACCTGCTGCGCGGCGCAACC  ATCAAGCCGTCGGTGCTGCAGGTGGAACACCACCCGTACCTGCAGCAGCCGCGCCTGATC  GAGTTCGCCCAGAGCCGCGGCATCGCCGTGACCGCCTACTCGAGCTTCGGCCCGCAGTCG  TTCGTGGAGCTGAACCAGGGCCGCGCCCTGAACACCAGCCCGCTGTTCGAGAACGAAACC  ATCAAGGCCATCGCCGCCAAGCACGGCAAGAGCCCGGCCCAGGTGCTGCTGCGCTGGTCG  AGCCAGCGCGGCATCGCCATCATCCCGAAGTCGAACACCGTGCCGCGCCTGCTGGAAAAC  AAGGACGTGAACAGCTTCGACCTGGACGAGCAGGACTTCGCCGACATCGCCAAGCTGGAC  ATCAACCTGCGCTTCAACGACCCGTGGGACTGGGACAAGATCCCGATCTTCGTGTAA |

**Table S3.** Upstream and downstream regions of *A0006* used to create *C. necator* H16 △*A0006.*

| **Description** | **5’ to 3’ sequence** |
| --- | --- |
| Upstream of *A0006* | CAAAGCTGGGCCGGATGCTGTGCGTGCTGCTGAAGGCGAAACGATACTGCTGTGGGACGG  TTCGAACGCTGGGGAATTCTTCAGGTCCAAGGTCGGGTTGGTTGCTTCGACGATGACGAA  GATTTCTCCGAGCAGTGTTTTTCGTCCGGCATACTTTTTTCATGTCGCCAAGCAAGCTGA  GCGATTCCTGAAGGCTCAAACCAATGGCACTGGCATTCCACACGTTGACCGAGAGCTTCT  CGAGGGGATAAAGGTCTTTTGTCCTGGCTCTACGGAGCAGCAATTACTTGCGGAAATCCT  CGACACTCTCGACACCGCCATCTACGAAACTGAAGCGATCATCGCCAAGCTCAAGGCGGT  CAAGCAAGGCCTGCTGCATGACCTCTTGACGCGCGGCATCGACGCCAACGGCGAATTGCG  CCCACCTCAGGCCGAGGCACCGCATCTCTACGAGTCGTCACCGTTGGGTTGGATTCCGAA  TGAGTGGGGTCTTGCTCCTACAGCAACTCGCTGCCATCTGATAACCAAAGGCACTACCCC  TGCGGCTAATGAGATGTGGCAGGGTGGCGCGGGAATTAGGTTTCTGCGAGTCGATAATCT  TTCTTTCGATGGACAACTGGATCTAGATGCAAGCACGTTTCGAGTTAGCCTTGCCACGCA  CAAAGGTTTTCTGGCTCGTTCAAGATGCCTTGAAGGTGATGTGCTGACGAACATCGTTGG  CCCACCTCTAGGGAAACTGGGGCTTGTTACCAAAGAAATTGGTGAGGTCAATATTAATCA  AGCAATTGCGTTATTTCGACCAACCGAACAACTACTGCCAAAGTTCCTATTAATCTGGCT  TAGTAGCTCAATCTCGCAGTCTTGGCTGAGGAACCGAGCCAAGCAGACGTCGGGACAAGT  GAATCTGACCCTCGCTCTATGCCAGGAGCTTCCTCTACCTCGGATGACGATCAATGAGCA  ACAGGCAATCGTTGACCGAGTTGATGCCGCGCAGGAACAAATCTGGTGTGAGGAGGAACT  GATCCGAAAGATGCGACTTGAGAAATCTGGCCTTATGGATGACCTCCTCACCGGCCGCGT  CCGCGTCAAGCCGCAGCTGGCGGAAACCAAACAAGCAGGGAGCGCCTGATG |
| Downstream of *A0006* | GCGCCTGATGCACGACAAGCTGATGCAGGTGTGATTCGATGCCTTCGCCGGTGAAATTGC  CAAGCCTTCAGATCGGTGACCTCCGGTTCACGCTCCAGCGGAGCGCGCGCCGCAGAACTA  TGCAGATCACCGTGGAGCGCAGTGGCGACTTGATGCTCTGCGCACCGCCGGAGGTGGACG  AGGCCGCGCTGCGAGCATTCGTGCTGGAGAAGCGCTTCTGGATCTACACCAAGCTGGCCG  AGAAGGACCGCTTGCAGCGCCAGGTTCCGCGCAAGGAATTCGTCGGAGGCGAGGGATTCT  TGTATCTCGGCCGCAGCCATCGGCTGAAGGTGGTCGATGAACAGAATGTGCCACTGAAGT  TGAATGGAGGCCGCTTTTGTCTGCGCCGTGACGCCCTACCCGCCGCGCGCGAGCATTTCA  TCCGCTGGTACGGCGAGCGTGCCAAGGCCTGGCTTTCGGGGCGTGTAGCTGACTACCAGT  CGCGAATGGAGGTGACGCCTGCCGGCGTCAAGGTGCAGGACCTTGGATATCGCTGGGGTT  CGTGTGGCAAGGGCGACTGGCTGTACTTCCACTGGAAGGCAATCCTGCTGCCGGCGCGCA  TCGCTGAGTATGTCGTGGTGCATGAGATTGCCCATCTGCATGAGCCGCACCACACGCCTG  CGTTCTGGCTTCGAGTGGAGCGTGCCATGCCGGACTATGCGCAACGCAAGGCCTGGCTGG  CCGAGCATGGAATCGATGTTGAAGGAATCTAAAGAACGATGGCTGACTATTTCACCAGTG  ACTACTTCAAGCTGCTGAACAAGTGGAAGGGGCAGAAGCGTGACGAGTCCAACCCCGAGC  AGAACCGCGCTTATGAAGATCTGAAGAAGGCCTACGAGGTGACGGAGGCGTGGGCGGACA  AGGTTAAGGCCGAGTTGTTCCCTGTCGGGCGCGTCGAGATTCGTAAGCGCCCGACCAACC  AGGGCAACAACTTTGCCAGCTACAACTGGGCCAAAATCTACCCTTCATCTGAGGCGCCGA  AAGAGTTGGCTTACACAGTTGGCATCGGCGCCGATGACGGCTTCGTAGTCAAGATCGATA  CCGTTGGGCTCGACGAATTGGACGCGACGAGAAAGGCCTAT |


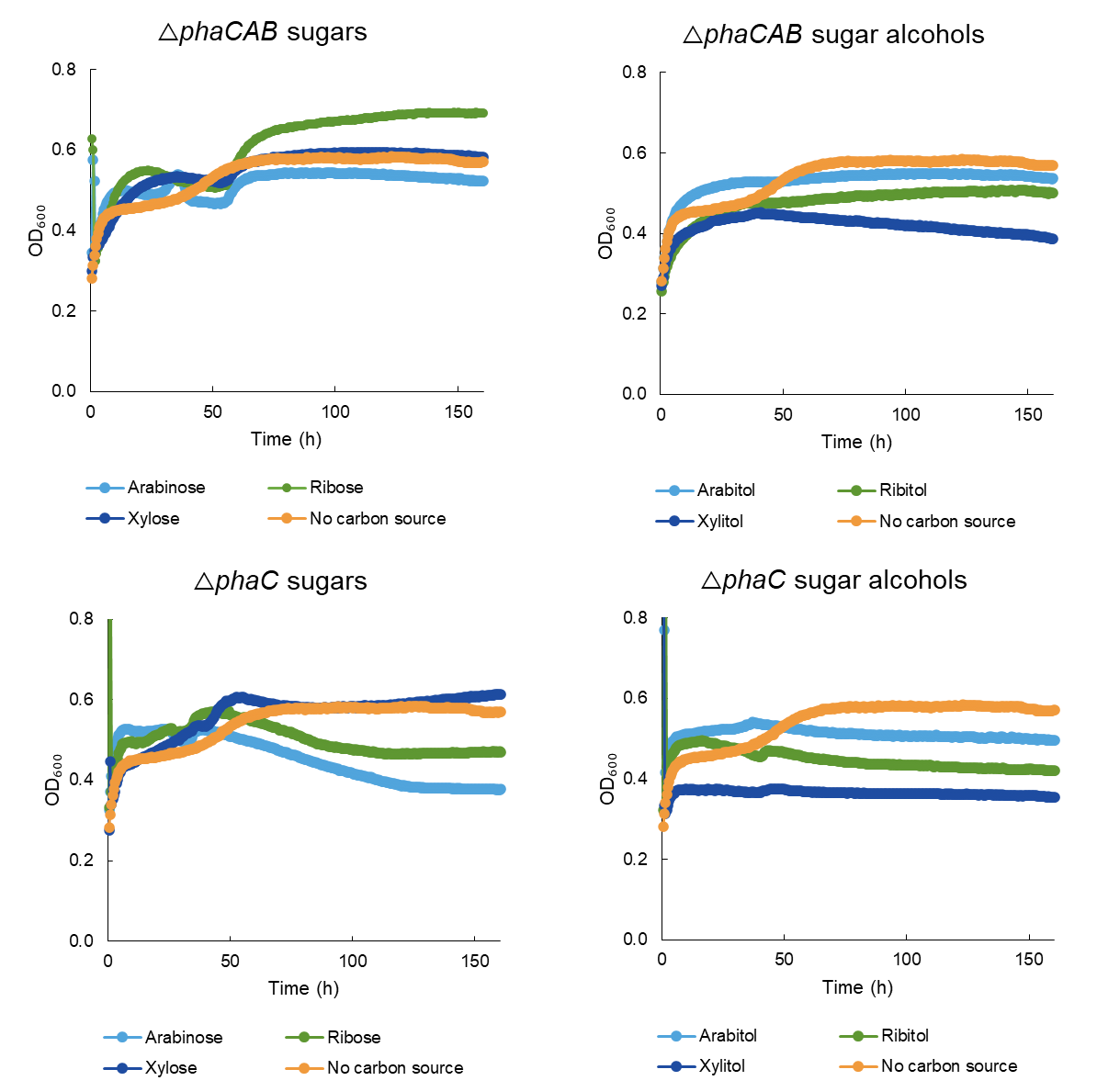


**Figure S1**. The growth of *C. necator* strains △*phaCAB* and △*phaC* on different sugars and sugar alcohols (100 mM). Precultures were grown in TSB. Cells were cultivated in 96-well plate at an initial OD_600_ of 0.05 in 130 μl of AUT media supplemented with the carbon sources. The cultures were topped with 50 μl of mineral oil to avoid evaporation. The negative control was cultivated in AUT media without any carbon source. The 96-well plate was incubated at 30°C using a double shake orbital at 282 rpm. All conditions had three replicates.


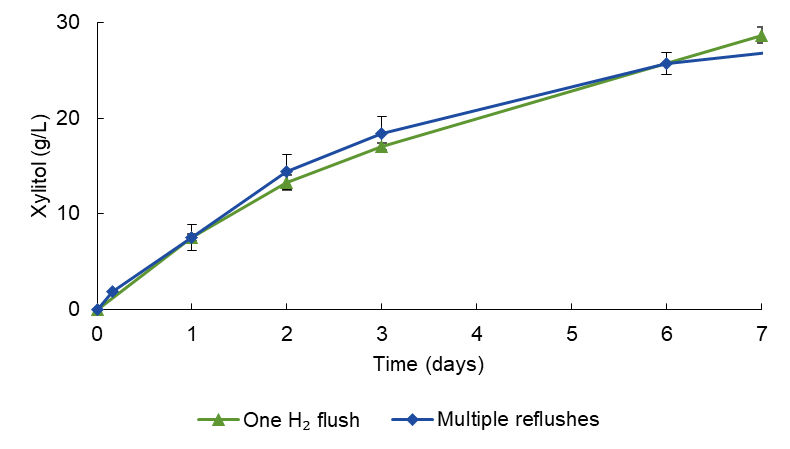


**Figure S2**. Comparison of bioconversion with a single H_2_ flush at the start and H_2_ flush after every sampling. The initial concentration of D-xylose was 34 g/L (one H_2_ flush) and 30 g/L (multiple reflushes) in 100 mM sodium phosphate buffer (pH 7). The cell concentrations were OD_600_ of 13 (one H_2_ flush) and OD_600_ of 17 (multiple reflushes).


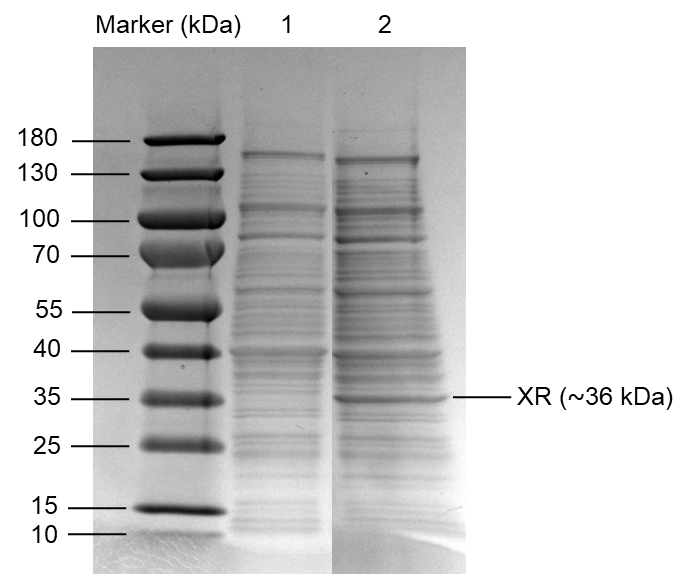


**Figure S3.** SDS-PAGE analysis of soluble extracts by *C. necator* H16 strains. Lane 1, wild type *C. necator* H16. Lane 2, △*phaCAB*_xr (△*phaCAB* with XRsti under Pj5 promoter).

**Full calculations**

**Hydrogen consumption**

The ideal gas law was used as a starting point for most of the calculations:

$$pV=nRT$$

Where p is the pressure (1 bar), V is the gas volume (L), n is the amount of gas (mol), R is the gas constant (0.08314 L bar K^-1^ mol^-1^), and T is the temperature (298.15 K).

The total volume of the serum bottle was 50 mL, with an initial liquid volume of 5 mL. Consequently, the volume of the gas in the bottle was 45 mL, and no overpressure was applied. The initial hydrogen amount in the serum bottles can be calculated:

$$n_{H_{2}\mathrm{initial}}=\frac{\mathrm{pV}}{\mathrm{RT}}= \frac{1 bar\times0.045 L}{0.083144 L bar \mathrm{mol}^{-1}K^{-1} \times298.15 K}=1.815 mmol$$

When H_2_ is consumed, only the pressure and the amount of hydrogen in the bottle change, as the gas volume is determined by the fixed volume of the bottle. However, when air is aspirated into the under-pressurized bottle, the volume of aspirated air is equal to the amount of hydrogen consumed. Consequently, the amount of hydrogen consumed can be estimated from the volume of air required to restore the pressure to atmospheric conditions. Additionally, sampling contributed to the under-pressure and must be accounted for:

$$V_{\mathrm{sampling}}=0.2 mL\times4=0.8 mL$$

The average aspirated volume into the serum flasks was 30 mL. The consumed H_2_ volume was calculated by subtracting the sampling volume:

$$V_{H_{2}\mathrm{consumed}}=30 mL-0.8 mL=29.2 mL$$

The amount of hydrogen consumed per bottle:

$$n_{H_{2}\mathrm{consumed}}=\frac{\mathrm{pV}}{\mathrm{RT}}= \frac{1 bar\times0.0292 L}{0.083144 L bar \mathrm{mol}^{-1}K^{-1} \times298.15 K}=1.178 mmol$$

The proportion of the initial H_2_ consumed per bottle is therefore:

$$Proportion of initial H_{2} consumed= \frac{n_{H_{2}\mathrm{consumed}}}{n_{H_{2}\mathrm{initial}}}\times100\%= \frac{1.178mmol}{1.815 mmol} \times100\%=65\%$$

**Xylitol production**

The amount of xylitol in the final samples was 46 g/L, which corresponds to 302 mM of xylitol. The final volume in the bottles was around 4.2 mL, considering that 0.8 mL was removed for sampling. Therefore, the final amount of xylitol in the serum bottles was:

$$n_{xylitol final}=0.302 M\times4.2 mL=1.268 mmol$$

Considering the xylitol in the samples, the total xylitol concentration produced was:

$$n_{xylitol total}= n_{xylitol final}+ n_{xylitol in samples} \approx1.3 mmol$$

Since some xylitol was produced without the presence of H_2_, this amount must be subtracted from the total to account for xylitol production with H_2_:

$$n_{xylitol produced with H_{2}}= n_{xylitol total}- n_{xylitol produced without H_{2}}=1.3 mmol-0.2 mmol=1.1 mmol$$

**Amount of H_2_ used for xylitol production from**

Since one mole of H_2_ is needed to reduce one mole of xylose, the amount of H_2_ used for xylitol production is 1.1 mmol. The proportion of H_2_ used for xylitol production is:

$$Proportion of H_{2} used for xylitol production= \frac{n_{H_{2}used for xylitol production}}{n_{H_{2}\mathrm{consumed}}}\times100\%= \frac{1.1 mmol}{1.178 mmol}\times100\%=93 \%$$

**Error considerations**

These calculations are estimates, and several factors may contribute to errors:

- The aspirated volume was measured using a 50 mL syringe, which lacks a fine-scale resolution.
- Sampling was performed with a 1 mL syringe, which also has limited scale resolution.
- Inaccurate sampling volumes can affect the total calculated amount of xylitol produced.
- The xylitol amount in samples is an average from multiple conversions.
- The xylitol amount which was produced without H_2_ is also an average from multiple conversions.
